# Relapse rate and predictors of relapse after cessation of glucocorticoid maintenance therapy in type 1 autoimmune pancreatitis: a multicenter retrospective study

**DOI:** 10.1186/s12876-023-02939-5

**Published:** 2023-09-04

**Authors:** Yusuke Kiyoshita, Yasutaka Ishii, Masahiro Serikawa, Keiji Hanada, Tamito Sasaki, Yoshifumi Fujimoto, Atsushi Yamaguchi, Ken Hirao, Bunjiro Noma, Tomoyuki Minami, Akihito Okazaki, Masanobu Yukutake, Teruo Mouri, Tomofumi Tsuboi, Yumiko Tatsukawa, Shinya Nakamura, Tetsuro Hirano, Juri Ikemoto, Sho Saeki, Yosuke Tamura, Sayaka Miyamoto, Masaru Furukawa, Kazuki Nakmura, Yumiko Yamashita, Noriaki Iijima, Shiro Oka

**Affiliations:** 1 Department of Gastroenterology, Graduate School of Biomedical & Health Sciences, Hiroshima University, 1-2-3 Kasumi, Minami-ku, Hiroshima, 734-8551 Japan; 2 Department of Gastroenterology, Onomichi General Hospital, Hiroshima, Japan; 3 Department of Gastroenterology, Hiroshima Prefectural Hospital, Hiroshima, Japan; 4 Department of Gastroenterology, Hiroshima General Hospital, Hiroshima, Japan; 5 Department of Gastroenterology, National Hospital Organization Kure Medical Center and Chugoku Cancer Center, Hiroshima, Japan; 6 Department of Internal Medicine, Hiroshima City Hiroshima Citizens Hospital, Hiroshima, Japan; 7 Department of Gastroenterology, Kure Kyosai Hospital, Hiroshima, Japan; 8 Department of Gastroenterology, Hiroshima Red Cross & Atomic-bomb Survivors Hospital, Hiroshima, Japan; 9 Department of Gastroenterology, National Hospital Organization Higashihiroshima Medical Center, Hiroshima, Japan; 10 Department of Gastroenterology, Hiroshima City North Medical Center Asa Citizens Hospital, Hiroshima, Japan; 11 Department of Gastroenterology, Chugoku Rosai Hospital, Hiroshima, Japan

**Keywords:** Autoimmune pancreatitis, Glucocorticoid maintenance therapy, Therapy cessation, Relapse

## Abstract

**Background:**

Type 1 autoimmune pancreatitis responds well to glucocorticoid therapy with a high remission rate. Moreover, glucocorticoid maintenance therapy can help prevent relapse. However, the relapse rate following cessation of long-term glucocorticoid therapy is unknown. The aim of this study was to clarify the relapse rate and predictors of relapse following long-term glucocorticoid therapy cessation.

**Methods:**

We analyzed 94 patients who achieved remission after undergoing glucocorticoid therapy, discontinued treatment after at least 6 months of maintenance therapy, and were subsequently followed up for at least 6 months. The patients were divided into three groups based on treatment duration (< 18, 18–36, and ≥ 36 months), and their relapse rates were compared. Univariate and multivariate analyses of clinical factors were conducted to identify relapse predictors.

**Results:**

After discontinuing glucocorticoid therapy, relapse was observed in 43 (45.7%) patients, with cumulative relapse rates of 28.2% at 1 year, 42.1% at 3 years, 47.0% at 5 years, and a plateau of 77.6% at 9 years. Of the 43 patients who relapsed, 25 (58.1%) relapsed within 1 year after after cessation of glucocorticoid therapy. Relapse and cumulative relapse rates did not differ significantly according to treatment duration. In the multivariate analysis, an elevated serum IgG4 level at the time of glucocorticoid cessation was found to be an independent predictor of relapse (hazard ratio, 4.511; *p* < 0.001).

**Conclusions:**

A high relapse rate occurred after cessation of glucocorticoid maintenance therapy, regardless of the duration of maintenance therapy, especially within the first year after cessation. However, the normalization of long-term serum IgG4 levels may be a factor in considering cessation.

## Background

Type 1 autoimmune pancreatitis (AIP) is a form of pancreatitis with suspected autoimmune mechanism involvement in its pathogenesis, and it is a suspected pancreatic manifestation of immunoglobulin G4 (IgG4)-related disease [1, 2]. Although the etiology is still unknown, AIP responds extremely well to glucocorticoid therapy with a high remission rate [3]. Furthermore, glucocorticoid maintenance therapy has been reported to be useful in curbing relapses, [4, 5] and the Japanese consensus guidelines recommend a 3-year treatment period [6]. However, the necessity and duration of maintenance therapy are controversial topics globally. Different glucocorticoid treatment protocols, including recommendations of no maintenance therapy or short-term maintenance therapy administered over a 6-month period, have been adopted in Europe and the United States, [7–9] as well as in South Korea [10.

In patients with AIP, relapse occurs relatively often, even during glucocorticoid therapy, [5, 11] although occurrence increases even more after glucocorticoid cessation. It has been reported that about half of patients treated with either no glucocorticoid maintenance therapy or short-term maintenance therapy relapsed in a relatively short period of time after discontinuing glucocorticoid [7, 8, 10]. On the other hand, Hirano et al. [12] reported that, among 21 patients who discontinued treatment after at least 3 years of glucocorticoid therapy, 10 (47.6%) relapsed after a mean follow-up period of 43 months. Even though relapse occurs often after long-term glucocorticoid therapy cessation in patients with AIP, studies on relapse rates and predictors after glucocorticoid cessation remain limited. The relapse rate for patients who received 1–3 years of glucocorticoid therapy, is completely unknown.

Although there is an international consensus [13] on the indications for glucocorticoid-based remission induction and maintenance therapies, no agreement has been reached on the criteria for discontinuing maintenance therapy. Furthermore, there has been limited research on predictors of relapse following cessation of maintenance therapy, and in clinical practice, the decision to discontinue maintenance therapy is often complicated.

This study aimed to determine the association between the duration of glucocorticoid maintenance therapy and relapse rate in patients with AIP who underwent at least 6 months of maintenance therapy and to determine the predictors of relapse following cessation of maintenance therapy.

## Methods

### Patients

We included 94 patients who achieved remission from a group of 254 patients treated with glucocorticoid therapy following the initial diagnosis of type 1 AIP at Hiroshima University Hospital and affiliated hospitals between January 2004 and December 2020. These 94 patients discontinued treatment after at least 6 months of maintenance therapy and were followed up for at least 6 months thereafter. The diagnosis of AIP was made according to the Japanese Clinical Diagnostic Criteria for AIP 2018^14^.

This study was conducted in accordance with the Declaration of Helsinki and approved by the ethics committee of Hiroshima University (approval number: E-246). Due to the retrospective nature of this study, the need for informed consent was waived.

### Treatment and follow-up strategies

As recommended by the Japanese consensus guidelines, [6] glucocorticoid therapy was indicated for patients with obstructive jaundice caused by bile duct stricture, those with abdominal and back pain, and those with extra-pancreatic lesions. Patients with endocrine or exocrine dysfunction or diffuse pancreatic enlargement were also treated with glucocorticoids.

Glucocorticoid therapy was performed according to the Japanese consensus guidelines [6, 14]. For remission induction therapy, the initial dose of oral prednisolone was approximately 0.6 mg/kg/day for 2–4 weeks. The dosage was then gradually tapered to a maintenance dose of 2.5–7.5 mg/day. The decision to discontinue maintenance therapy was made by the attending physician after imaging studies (such as computed tomography [CT] and magnetic resonance imaging [MRI]) confirmed complete remission of IgG4-related diseases, including AIP, and with reference to serum IgG4 levels. For timeously detection of relapse after cessation of maintenance therapy, among the imaging studies including CT, MRI, ultrasonography, and endoscopic ultrasonography, mainly CT or MRI was typically performed every 6 months. Furthermore, serum IgG4 level measurements and hematological chemistry tests were performed every 3–6 months. When patients presented with clinical symptoms, such as abdominal pain, back pain, and jaundice, or when elevated hepatobiliary or pancreatic enzymes were detected, additional imaging were performed on an ad hoc basis.

### Definitions

AIP relapse was defined as follows: reswelling of the pancreas or re-narrowing of the main pancreatic duct, development, or reappearance of extra-pancreatic lesions, such as extra-pancreatic sclerosing cholangitis, retroperitoneal fibrosis, sclerosing dacryoadenitis/sialadenitis, or renal lesions on imaging studies, regardless of serum IgG4 elevation. Sclerosing cholangitis was evaluated using endoscopic retrograde cholangiopancreatography or magnetic resonance cholangiopancreatography. Other extra-pancreatic lesions were evaluated using CT. Elevated serum IgG4 levels were defined as ≥ 135 mg/dL according to the Japanese Clinical Diagnostic Criteria for AIP [15.

### Outcomes

The primary endpoint was the relapse rate following glucocorticoid therapy cessation. In addition to the overall relapse rate, patients were divided into three groups according to the duration of glucocorticoid treatment: < 18 months (short-term group), 18–36 months (medium-term group), and ≥ 36 months (long-term group). Secondary endpoints were predictors of relapse following glucocorticoid therapy cessation. The multivariate analysis included the following variables: age, sex, elevated serum IgG levels at the time of glucocorticoid cessation, elevated serum IgG4 levels at the time of glucocorticoid cessation, diffuse pancreatic enlargement, diffuse narrowing of the main pancreatic duct, presence of extra-pancreatic lesions (extra-pancreatic sclerosing cholangitis, sclerosing dacryoadenitis/sialadenitis, retroperitoneal fibrosis, or renal lesions), maintenance dose of prednisolone before cessation < 5 mg/day, a duration of glucocorticoid administration of ≥ 3 years.

### Statistical analyses

Statistical analyses were performed using JMP Pro 16.0.0 (SAS Institute Inc. Cary, North Carolina, USA). The Bonferroni adjustment method for multiple comparisons were used to compare the clinical factors among the three groups, based on duration of steroid therapy. The relapse rates were calculated using the Kaplan–Meier method, and the log-rank test was used to compare them. The Cox proportional hazards model was used to perform univariate and multivariate analyses of relapse predictors. Parameters with a *p* value < 0.1 in the univariate analysis were included in the multivariate analysis. *P* values < 0.05 were considered statistically significant.

## Results

### Patient characteristics

The clinical characteristics of the 94 patients with type 1 AIP are shown in Table 1. The median serum IgG4 level at the time of AIP diagnosis was 332 (interquartile range [IQR], 161–557) mg/dL, and elevated serum IgG4 levels (≥ 135 mg/dL) were observed in 79 (84.0%) patients. In contrast, the median serum IgG4 level at the time of glucocorticoid cessation was 147 (IQR, 80–233) mg/dL, and elevated serum IgG4 levels were observed in 55 (58.5%) patients. Extra-pancreatic lesions were observed in 27 (28.7%) patients, and only one patient had multiple extra-pancreatic lesions, sclerosing dacryoadenitis/sialadenitis and renal lesions. There were no patients in which glucocorticoid-sparing agents, immune-modulators and rituximab, were used.

**Table 1:** Clinical profiles of the 94 patients with type 1 AIP

Characteristics	Values
Age at diagnosis (years)	68 (60–71)
Age at glucocorticoid cessation (years)	70 (63–74)
Sex (male to female)	76 : 18
Serological findings at diagnosis	
Serum IgG level (mg/dL)	1613 (1442–2102)
Elevated serum IgG level (≥ 1800 mg/dL), n (%)	35 (37.2%)
Serum IgG4 level (mg/dL)	332 (161–557)
Elevated serum IgG4 level (≥ 135 mg/dL), n (%)	79 (84.0%)
Serological findings at the time of glucocorticoid cessation	
Serum IgG level (mg/dL)	1188 (1020–1336)
Elevated serum IgG level (≥ 1800 mg/dL), n (%)	12 (12.8%)
Serum IgG4 level (mg/dL)	147 (80–233)
Elevated serum IgG4 level (≥ 135 mg/dL), n (%)	55 (58.5%)
Enlargement of the pancreas, n (%)	94 (100%)
Diffuse	39 (41.5%)
Segmental/focal	55 (58.5%)
Irregular narrowing of the MPD, n (%)	94 (100%)
Diffuse or multiple	37 (39.4%)
Segmental/focal	57 (60.6%)
Extra-pancreatic lesion, n (%)	27 (28.7%)
Extra-pancreatic sclerosing cholangitis	9 (9.6%)
Sclerosing dacryoadenitis & sialadenitis	10 (10.6%)
Retroperitoneal fibrosis	7 (7.4%)
Renal lesion	2 (2.1%)
Diagnosis according to the Japanese diagnostic criteria, n (%)	
Definite	78 (83.0%)
Probable	5 (5.3%)
Possible	11 (11.7%)
Duration of glucocorticoid therapy (months)	24 (14–37)
Maintenance dose of prednisolone before cessation (mg/day)	5 (2.5–5)
Follow-up period from glucocorticoid cessation (months)	42 (20–82)
Follow-up period from diagnosis (months)	82 (50–118)

Data are expressed as number (percentage) or median (interquartile range).

AIP, autoimmune pancreatitis; MPD, main pancreatic duct

The short-term glucocorticoid treatment group comprised 34 patients; the medium-term group, 30; and the long-term group, 30. The clinical characteristics of each group are shown in Table 2. There were no significant differences among the three groups in age at the time of glucocorticoid cessation; sex; serum IgG4 levels at time of diagnosis or time of glucocorticoid cessation; frequency of diffuse pancreatic enlargement, diffuse irregular main pancreatic duct narrowing, or extra-pancreatic lesions; maintenance dose of prednisolone before cessation; and median follow-up period after glucocorticoid cessation. Elevated serum IgG4 levels at diagnosis were observed significantly less frequently in the short-term group than in the medium- and long-term groups (*p* = 0.021 and 0.005, respectively). Four patients (4.3%) experienced glucocorticoid side effects or medical occurrences suspected to be side effects, namely femoral neck fracture, pulmonary tuberculosis, acute myocardial infarction, and angina pectoris.

**Table 2:** Comparison of clinical factors based on the duration of glucocorticoid therapy

Characteristics	< 18 months (n = 34)	18–36 months (n = 30)	≥ 36 months (n = 30)	*P* value
Age at glucocorticoid cessation (years)	70 (61–73)	70 (61–73)	71 (65–80)	0.279
Sex (male), n (%)	27 (79.4%)	22 (73.3%)	27 (90.0%)	0.251
Serological findings at diagnosis				
Serum IgG level (mg/dL)	1586 (1441–1965)	1664 (1467–2257)	1726 (1374–2105)	0.667
Elevated serum IgG level (≥ 1800 mg/dL), n (%)	9 (26.5%)	13 (43.3%)	13 (43.3%)	0.267
Serum IgG4 level (mg/dL)	238 (120–506)	406 (184–573)	316 (174–571)	0.204
Elevated serum IgG4 level (≥ 135 mg/dL), n (%)	22 (64.7%)	28 (93.3%)	29 (96.7%)	< 0.001
Serological findings at the time of glucocorticoid cessation				
Serum IgG level (mg/dL)	1112 (1000–1304)	1198 (1026–1287)	1267 (1076–2099)	0.170
Elevated serum IgG level (≥ 1800 mg/dL), n (%)	3 (8.8%)	2 (6.7%)	7 (23.3%)	0.106
Serum IgG4 level (mg/dL)	139 (41–203)	147 (91–232)	172 (100–412)	0.101
Elevated serum IgG4 level (≥ 135 mg/dL), n (%)	17 (50.0%)	19 (63.3%)	19 (63.3%)	0.452
Diffuse enlargement of the pancreas, n (%)	12 (35.3%)	12 (40.0%)	15 (50.0%)	0.482
Diffuse irregular narrowing of the MPD, n (%)	11 (32.4%)	11 (36.7%)	15 (50.0%)	0.331
Extra-pancreatic lesion, n (%)	11 (32.4%)	9 (30.0%)	7 (23.3%)	0.716
Maintenance dose of prednisolone before cessation (mg/day)	5 (2.5–5)	5 (2.5–5)	4 (2.5–5)	0.364
Follow-up period from glucocorticoid cessation (months)	65 (23–87)	52 (25–85)	31 (13–74)	0.198

Data are expressed as number (percentage) or median (interquartile range).　MPD, main pancreatic duct

### Relapse rate

**Fig. 1:**
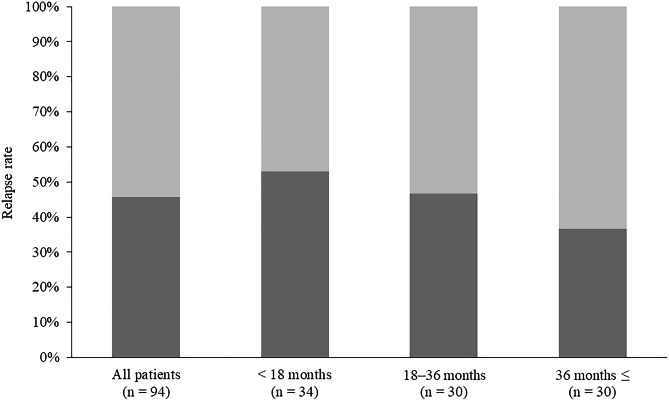
Relapse rate of patients with type 1 autoimmune pancreatitis after cessation of glucocorticoid therapy The entire patient group was divided into three subgroups according to glucocorticoid therapy duration as follows: < 18 months (short-term group), 18–36 months (medium-term group), and ≥ 36 months (long-term group)

**Fig. 2:**
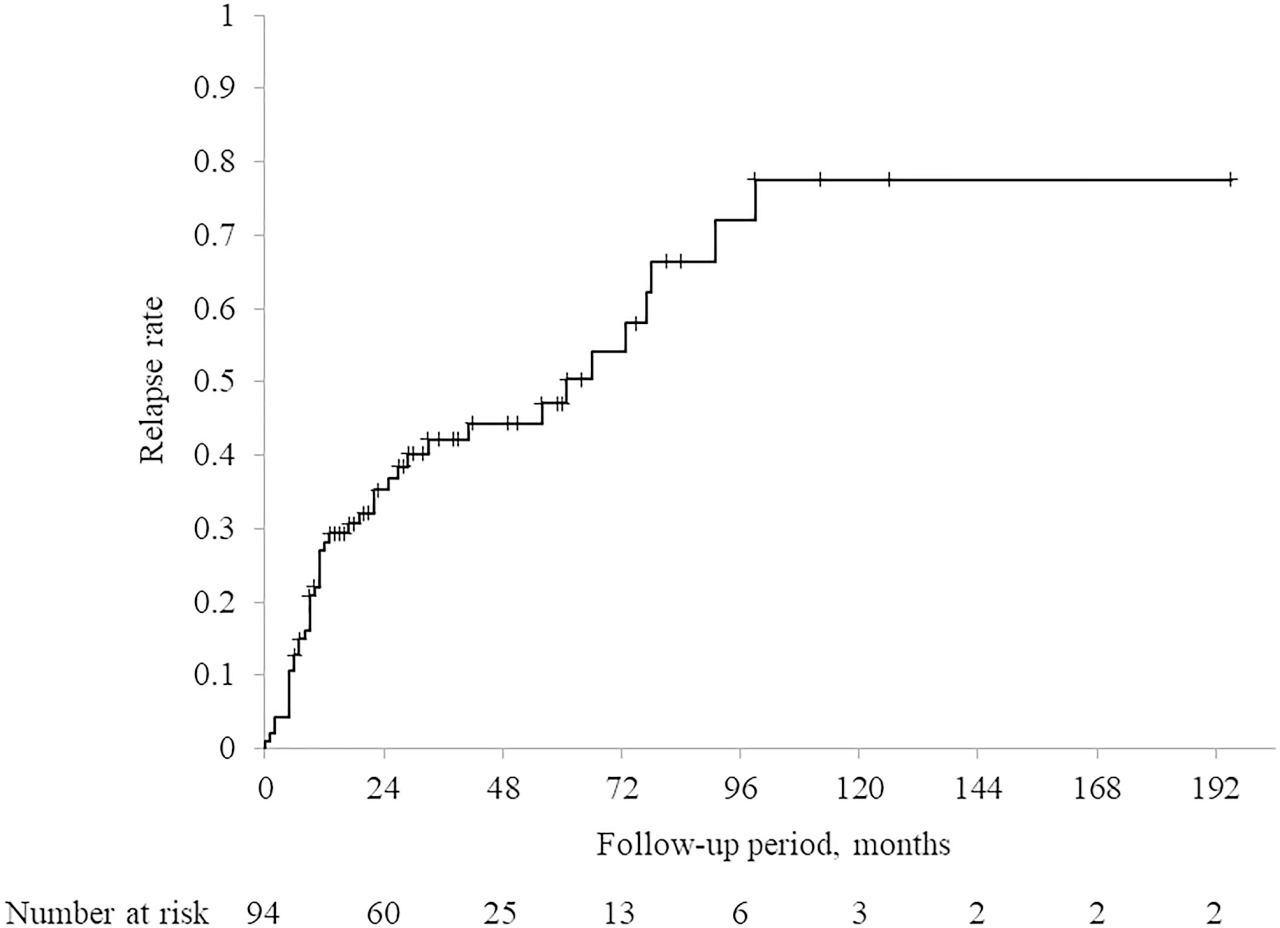
Kaplan-Meier analysis of the relapse rate for the entire group

**Fig. 3:**
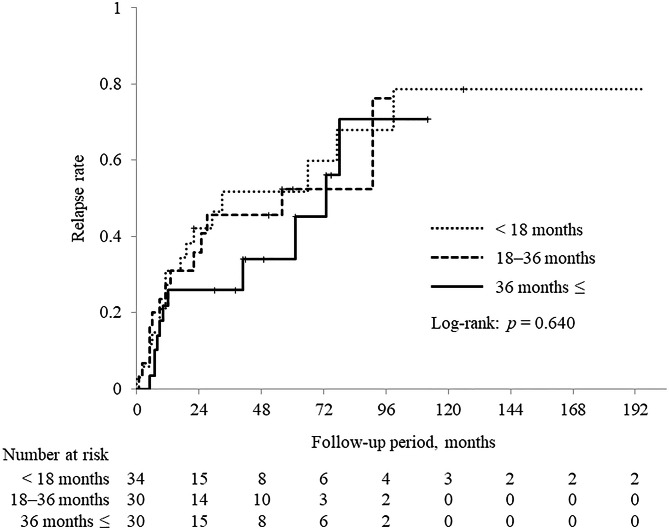
Kaplan-Meier analysis of the relapse rate according to the duration of glucocorticoid therapy The cumulative relapse rate was compared using the log-rank test

Relapse rates after glucocorticoid cessation are shown in Fig. 1. Relapse occurred in 43 (45.7%) of the 94 patients. The sites of recurrence were the pancreas in 34 patients, the extra-pancreatic bile duct in 5, the lacrimal/salivary gland in 3, the retroperitoneum in 6, and the kidney in 3. Furthermore, the sites of recurrence were solely intra-pancreatic in 27 (62.8%) patients, solely extra-pancreatic in 9 (20.9%), and both intra- and extra-pancreatic in 7 (16.3%). Of the 43 patients who relapsed, 25 (58.1%) relapsed within 1 year, 30 (69.8%) within 2 years, and 34 (79.1%) within 3 years. Relapse rates according to the duration of glucocorticoid treatment were as follows: 52.9% (18/34) in the short-term group, 46.7% (14/30) in the medium-term group, and 36.7% (11/30) in the long-term group, with no significant difference. The Kaplan-Meier analysis of the relapse rates for the entire group (94 patients) is shown in Fig. 2. The cumulative relapse rate was 28.2% at 1 year, 42.1% at 3 years, 47.0% at 5 years, 66.4% at 7 years, and a plateau of 77.6% at 9 years following glucocorticoid cessation. Then, the Kaplan-Meier analysis of the relapse according to the duration of glucocorticoid treatment is shown in Fig. 3. The median relapse-free time were as follows: 33 months in the short-term group, 56 months in the medium-term group, and 73 months in the long-term group. There was no significant difference in cumulative relapse rates among the three groups (log-rank: *p* = 0.640).

### Predictive factors of relapse after glucocorticoid therapy cessation

The results of univariate and multivariate analyses, which were performed to identify useful predictors of relapse after glucocorticoid therapy cessation, are shown in Table 3. The univariate analysis revealed that elevated serum IgG4 levels at the time of glucocorticoid cessation and multiple extra-pancreatic lesions were significantly associated with relapse (*p* = < 0.001 and 0.029, respectively). We performed multivariate analysis of these three factors, and since the univariate analysis of diffuse pancreatic enlargement and diffuse/multiple irregular narrowing of the main pancreatic duct showed *p* < 0.1, we included these in the multivariate analysis as well. The multivariate analysis showed that elevated serum IgG4 levels at the time of glucocorticoid cessation were an independent predictor of relapse (hazard ratio: 4.511, 95% confidence interval: 2.069–9.834, *p* < 0.001).

**Table 3:** Univariate and multivariate analyses of the predictors for relapse after glucocorticoid cessation

	Relapse	Non-relapse	Univariate analysis			Multivariate analysis	
Parameters	(n = 43)	(n = 51)	Hazard ratio (95% CI)	*P* value		Hazard ratio (95% CI)	*P* value
Age at glucocorticoid cessation ≥ 70 years	18	33	0.706 (0.379–1.314)	0.272			
Sex (male)	35	41	1.133 (0.519–2.470)	0.754			
Serum IgG level ≥ 1800 mg/dL at the time of glucocorticoid cessation	7	5	1.245 (0.553–2.800)	0.597			
Serum IgG4 level ≥ 135 mg/dL at the time of glucocorticoid cessation	35	20	4.045 (1.873–8.734)	< 0.001		4.511 (2.069–9.834)	< 0.001
Diffuse enlargement of the pancreas	14	25	0.562 (0.297–1.065)	0.077		0.627 (0.239–1.646)	0.344
Diffuse or multiple irregular narrowing of the MPD	12	25	0.560 (0.287–1.091)	0.088		0.679 (0.250–1.848)	0.449
Extra-pancreatic sclerosing cholangitis	6	3	1.637 (0.682–3.932)	0.270			
Sclerosing dacryoadenitis/sialadenitis	5	5	1.153 (0.452–2.940)	0.766			
Retroperitoneal fibrosis	3	4	0.810 (0.249–2.629)	0.726			
Renal lesion	1	1	3.942 (0.514–30.25)	0.187			
Multiple extra-pancreatic lesions	1	0	10.01 (1.268–79.00)	0.029		4.385 (0.605–38.86)	0.137
Maintenance dose of prednisolone before cessation < 5 mg/day	23	16	1.300 (0.712–2.377)	0.393			
Duration of glucocorticoid administration ≥ 3 years	11	19	0.722 (0.363–1.435)	0.353			
MPD, main pancreatic duct; CI, confidence interval							

## Discussion

Although type 1 AIP responds well to glucocorticoids, with the possibility of a high remission rate, complete consensus has not been reached on the duration of maintenance therapy, timing of glucocorticoid cessation, and causes of the high relapse rate after cessation. In this study of 94 patients with type 1 AIP on glucocorticoid maintenance therapy for at least 6 months, with a median follow-up period of 42 months, the relapse rate after glucocorticoid cessation was 45.7%. The cumulative relapse rates were also high. Similar to this study, a study by a South Korean research team, whose protocol included a short 6-month maintenance therapy period, reported a cumulative relapse rate of 10.9% at 1 year, 37.2% at 3 years, 44.8% at 5 years, 61.9% at 7 years, and a plateau of 70.2% at 8 years, after the initiation of treatment [10]. In contrast, studies from two centers from the United States and Europe, [7, 8] where maintenance therapy were not included in their protocols, reported that > 50% of cases relapsed 3 months after glucocorticoid cessation. Although direct comparisons are difficult due to differences in the definition of relapse and number of cases, the findings suggest that glucocorticoid maintenance therapy, administered over a certain period of time, may be useful in curbing short-term relapses after cessation.

In this study, the patients were divided into three groups based on the duration of glucocorticoid treatment, and relapse rates were examined. However, no significant difference was found in the cumulative relapse rates among the three groups. The 3-year glucocorticoid treatment period, which is recommended by the Japanese consensus guidelines, [6] is based on the results of a multicenter, retrospective study that reported a 92% cumulative relapse rate at 3 years in patients treated with glucocorticoids [16]. However, this does not imply that 3 years of glucocorticoid treatment, including maintenance therapy, will reduce the relapse rate after cessation; even after long-term glucocorticoid treatment, a high relapse rate still exists. Hirano et al. [12] recommended continuing glucocorticoid maintenance therapy beyond 3 years to prevent relapse; however, there are some reports that the incidence of serious adverse events (severe infections, fractures, osteonecrosis of the femoral head, or cardiovascular disease) rises with increasing cumulative glucocorticoid doses, [4, 17] and caution is advised with long-term maintenance therapy. Hart et al. [3] reported a high incidence of pancreatic stones in patients who relapsed, suggesting that repeated relapses may worsen pancreatic function. However, no research on the impact of relapse on life prognosis has been published, and to determine the optimal duration of glucocorticoid therapy, it is necessary to clarify this impact as well.

Of the relapse cases included in this study, 58.1% (25/43) relapsed within 1 year of glucocorticoid cessation. The Kaplan-Meier curve for the relapse rate showed a rapid increase in the relapse rate within approximately 1 year of glucocorticoid cessation, regardless of the duration of glucocorticoid treatment. A study by a South Korean research team [10] also found that the majority of relapses occurred within 2 years of treatment initiation, suggesting that careful follow-up is needed for approximately 1 year after cessation, with special attention to relapse. However, research has shown that it takes 8 years for the cumulative relapse rate to reach a plateau after glucocorticoid cessation, and long-term follow-up is thus also required. However, guidelines do not describe follow-up protocols or intervals to implement during or after glucocorticoid therapy. Regular serum IgG4 level measurement is necessary because persistently high blood IgG4 levels [16] after initiation of glucocorticoid therapy and a rebound during the course of the disease [18] have been reported to be predictors of relapse. In addition, hepatobiliary enzyme, serum bilirubin, and pancreatic enzyme levels should be measured, since elevated levels are associated with sclerosing cholangitis and intra-pancreatic recurrences. Although jaundice and abdominal pain are frequent clinical manifestations of AIP, it is reported that patients convey no subjective symptoms in nearly 40% of cases, and the diagnosis is reached inadvertently based on imaging, abnormal blood tests, or onset or exacerbation of diabetes [19]. Therefore, imaging will also play a role in confirming relapse. A nationwide survey in Japan reported that 49.7% of recurrence sites were intra-pancreatic, 30.5% were extra-pancreatic, and 19.8% were both intra- and extra-pancreatic [19]. In this study, 62.8% of the recurrence sites were solely intra-pancreatic, 20.9% were solely extra-pancreatic, and 16.3% were both intra- and extra-pancreatic. To confirm relapse, systemic imaging, such as CT, should be performed to assess extra-pancreatic lesions.

In addition to short-term glucocorticoid therapy cessation, other predictors of relapse include high serum IgG4 levels at diagnosis, 20] persistently high or elevated serum IgG4 levels after glucocorticoid induction therapy, [16, 18] proximal bile duct stricture, [3, 9] diffuse pancreatic enlargement, [4] and extra-pancreatic lesions [11, 21, 22]. The present study showed that elevated serum IgG4 levels (≥ 135 mg/dL) at the time of glucocorticoid cessation were an independent predictor of relapse in patients who discontinued therapy after at least 6 months of maintenance. There is limited research on the predictors of relapse in patients who discontinued glucocorticoids after maintenance therapy. It has been reported that proximal bile duct stricture is a predictor of relapse in patients who discontinued glucocorticoid therapy after a short 6-month maintenance period [10]. In this study, extra-pancreatic sclerosing cholangitis was not found to be a significant predictor of relapse, although the number of patients with extra-pancreatic bile duct stricture was small (9; 9.6%); therefore, further review is necessary to determine the predicting factors of relapse. In this study, 8 of the 43 patients who relapsed after cessation had normal serum IgG4 levels (< 135 mg/dL) at the time of glucocorticoid cessation, and 2 of the 8 patients did not have elevated serum IgG4 levels at the time of AIP diagnosis as well. In patients with AIP without elevated serum IgG4 levels, serum IgG4 levels might not be as useful in the decision to discontinue glucocorticoids. Fukiage et al. reported that serum autotaxin was useful for early detection of relapse in a male patient with type 1 AIP [23]. In the future, it may be necessary to explore biomarkers other than serum IgG4 that are useful for prediction and early detection of relapse.

There are some limitations to this study. First, this was a retrospective study. Second, since the timing of glucocorticoid maintenance therapy cessation was at the discretion of the attending physician, there may have been some bias in patient selection. The short-term group contained significantly more patients with serum IgG4 levels in the normal range at the time of AIP diagnosis than the other two groups, which may have influenced the duration of maintenance therapy. Third, although the sample size of this study was considerably larger than that of previous studies on relapse after cessation of glucocorticoid therapy, it remains insufficient. However, AIP is a relatively rare condition, [19] which makes the possibility of larger studies difficult. Future prospective, multicenter studies are needed to investigate relapse rates, according to duration of maintenance therapy and predictors of relapse following glucocorticoid cessation.

## Conclusions

In this study, a high relapse rate occurred after cessation of glucocorticoid maintenance therapy, regardless of the duration of maintenance therapy, especially within the first year after cessation. Furthermore, elevated serum IgG4 at the time of glucocorticoid cessation was an independent and significant predictor of relapse after cessation. Therefore, if glucocorticoid treatment has resulted in prolonged remission, evidenced by imaging and normalization of serum IgG4 levels, cessation may be considered in individual cases without regard to the duration of maintenance therapy. However, regular follow-up is essential after treatment discontinuation while keeping relapse in mind.

## Data Availability

The datasets used and analyzed during the current study are available from the corresponding author on reasonable request.

## Abbreviations

AIP: Autoimmune pancreatitis
IgG4: Immunoglobulin G4
CT: Computed tomography
MRI: Magnetic resonance imaging

## References

[1] Stone JH, Khosroshahi A, Deshpande V (2012). Recommendations for the nomenclature of IgG4-related disease and its individual organ system manifestations. Arthritis Rheum.

[2] Kamisawa T, Zen Y, Pillai S (2015). IgG4-related disease. Lancet.

[3] Hart PA, Kamisawa T, Brugge WR (2013). Long-term outcomes of autoimmune pancreatitis: a multicentre, international analysis. Gut.

[4] Kubota K, Kamisawa T, Okazaki K (2017). Low-dose maintenance steroid treatment could reduce the relapse rate in patients with type 1 autoimmune pancreatitis: a long-term japanese multicenter analysis of 510 patients. J Gastroenterol.

[5] Masamune A, Nishimori I, Kikuta K (2017). Randomized controlled trial of long-term maintenance corticosteroid therapy in patients with autoimmune pancreatitis. Gut.

[6] Okazaki K, Kawa S, Kamisawa T (2022). Amendment of the japanese consensus guidelines for autoimmune pancreatitis, 2020. J Gastroenterol.

[7] Ghazale A, Chari ST, Zhang L (2008). Immunoglobulin G4-associated cholangitis: clinical profile and response to therapy. Gastroenterology.

[8] Raina A, Yadav D, Krasinskas AM (2009). Evaluation and management of autoimmune pancreatitis: experience at a large US center. Am J Gastroenterol.

[9] Löhr JM, Beuers U, Vujasinovic M (2020). European Guideline on IgG4-related digestive disease - UEG and SGF evidence-based recommendations. United Eur Gastroenterol J.

[10] Lee HW, Moon SH, Kim MH (2018). Relapse rate and predictors of relapse in a large single center cohort of type 1 autoimmune pancreatitis: long-term follow-up results after steroid therapy with short-duration maintenance treatment. J Gastroenterol.

[11] Ishii Y, Serikawa M, Sasaki T (2019). Impact of sclerosing dacryoadenitis/sialadenitis on relapse during steroid therapy in patients with type 1 autoimmune pancreatitis. Scand J Gastroenterol.

[12] Hirano K, Tada M, Isayama H (2016). Outcome of long-term maintenance steroid therapy cessation in patients with autoimmune pancreatitis: a prospective study. J Clin Gastroenterol.

[13] Okazaki K, Chari ST, Frulloni L (2017). International consensus for the treatment of autoimmune pancreatitis. Pancreatology.

[14] Kamisawa T, Okazaki K, Kawa S (2014). Amendment of the japanese consensus guidelines for autoimmune pancreatitis, 2013 III. Treatment and prognosis of autoimmune pancreatitis. J Gastroenterol.

[15] Kawa S, Kamisawa T, Notohara K (2020). Japanese clinical diagnostic criteria for autoimmune pancreatitis, 2018: revision of japanese clinical diagnostic criteria for autoimmune pancreatitis, 2011. Pancreas.

[16] Kamisawa T, Shimosegawa T, Okazaki K (2009). Standard steroid treatment for autoimmune pancreatitis. Gut.

[17] Shimizu S, Naitoh I, Nakazawa T (2015). Correlation between long-term outcome and steroid therapy in type 1 autoimmune pancreatitis; relapse, malignancy and side effect of steroid. Scand J Gastroenterol.

[18] Suzuki D, Shimizu K, Tokushige K (2018). Relative rise of serum IgG4 levels after steroid therapy for autoimmune pancreatitis predicts the likelihood of relapse. Pancreas.

[19] Masamune A, Kikuta K, Hamada S (2020). Nationwide epidemiological survey of autoimmune pancreatitis in Japan in 2016. J Gastroenterol.

[20] Kubota K, Watanabe S, Uchiyama T, Kato S, Sekino Y, Suzuki K (2011). Factors predictive of relapse and spontaneous remission of autoimmune pancreatitis patients treated/not treated with corticosteroids. J Gastroenterol.

[21] Kubota K, Kamisawa T, Hirano K (2018). Clinical course of type 1 autoimmune pancreatitis patients without steroid treatment: a japanese multicenter study of 97 patients. J Hepatobiliary Pancreat Sci.

[22] Nakamura A, Ozawa M, Watanabe T (2018). Predictive factors for autoimmune pancreatitis relapse after 3 years of maintenance therapy. Pancreas.

[23] Fukiage A, Fujino H, Miki D (2021). Clinical usefulness of serum autotaxin for early prediction of relapse in male patients with type 1 autoimmune pancreatitis. Dig Dis Sci.

